# The impact of Aurora kinase A genetic polymorphisms on cervical cancer progression and clinicopathologic characteristics

**DOI:** 10.7150/ijms.58516

**Published:** 2021-04-22

**Authors:** Pei-Ju Wu, Chun-Hao Wang, Ming-Hong Hsieh, Chung-Yuan Lee, Po-Hui Wang, Chiao-Wen Lin, Shun-Fa Yang, Maw-Sheng Lee

**Affiliations:** 1 Institute of Medicine, Chung Shan Medical University, Taichung, Taiwan.; 2 School of Medicine, Chung Shan Medical University, Taichung, Taiwan.; 3 Department of Obstetrics and Gynecology, Chung Shan Medical University Hospital, Taichung, Taiwan.; 4 Department of Medicine, National Taiwan University, Taipei, Taiwan.; 5 Department of Psychiatry, Chung Shan Medical University Hospital, Taichung, Taiwan.; 6 Department of Obstetrics and Gynecology, Chiayi Chang Gung Memorial Hospital, Chiayi, Taiwan.; 7 Department of Nursing, Chang Gung University of Science and Technology, Chiayi Campus, Chiayi, Taiwan.; 8 Institute of Oral Sciences, Chung Shan Medical University, Taichung, Taiwan.; 9 Department of Medical Research, Chung Shan Medical University Hospital, Taichung, Taiwan.

**Keywords:** Aurora kinase A, single nucleotide polymorphisms, uterine cervical cancer, pelvic lymph node metastasis

## Abstract

The aims of this study were to explore the involvement of Aurora kinase A (AURKA) gene single nucleotide polymorphisms (SNPs) in uterine cervical cancer that has not yet been investigated. One hundred and six patients with cervical invasive cancer and 94 patients with precancerous lesions, and 302 Taiwanese female individuals were included. AURKA SNPs rs2273535, rs6024836, rs2064863 and rs1047972 were analyzed for genotypic distributions using real-time polymerase chain reaction. There were no statistically significant differences in the genetic frequencies of AURKA SNPs among patients with invasive cancer and those with precancerous lesions of uterine cervix and control women. There were no associations among AURKA SNPs and clinicopathologcal variables and recurrence and survival events. However, in a multivariate analysis, cervical cancer patients with adenocarcinoma (HR: 3.18, 95% CI: 1.23-8.23; p=0.017) and larger tumor (HR: 5.61, 95% CI: 2.10-14.95; p=0.001) had poorer recurrence-free survival. In conclusion, tumor size and pelvic lymph node status rather than AURKA SNPs were the most obvious independent parameter that could significantly predict 5 years survival rate in Taiwanese women with cervical cancer.

## Introduction

Uterine cervical cancer still remains an important public health issue, leading to a considerable morbidity and mortality rate 1, 2. It is the eighth most common malignant tumor among women and the second most common gynecological malignancy in Taiwan. According to the Taiwan Annual Cancer Registry Report it revealed the age-standardized incidence rate of cervical cancer in 2014 to be 8.50 per 100000 women from the Health Promotion Administration of the Ministry of Health and Welfare. The age-standardized mortality rate of cervical cancer was 3.39 per 100000 women in 2014, which was the seventh leading cause of cancer deaths among women in Taiwan.0

A continuous and multi-step process is required for the formation of invasive cancer of uterine cervix. The multi-step process consists of neoplastic transformation from cervical intraepithelial neoplasia (CIN), known as precancerous lesions, to invasive cancer, which was regarded as the end of CIN progression 3, 4. When mitoses and immature cells occupy in the lower one-third of cervical epithelium, it is referred to CIN 1 histologically (also regarded as low-grade CIN or dysplasia, or mild dysplasia) or low-grade squamous cell intraepithelial lesions (LSIL), for cytological counterpart. When mitoses and immature cells progress in the middle and upper third epithelium, CIN 2 (moderate dysplasia) and CIN 3 (severe dysplasia and carcinoma *in situ* when whole epithelium is involved) are denominated histologically, respectively. They are collectively regarded as high-grade CIN or high-grade dysplasia and considered as precancerous lesions. The cytological counterpart is called high-grade squamous cell intraepithelial lesions (HSIL) 5.

Aurora kinase A (AURKA) is located at chromosome 20q13.2 and belongs to Aurora kinases, a family, which consists of Aurora kinase A, B (AURKB) and C (AURKC) 6, 7. It functions as a check-point-associated kinase in the cell cycle, and is linked to the regulation of transition from G2 to M phase in cell division 7-9. In addition to focusing on cell division, it has been reported to also regulate the self-renewal and reprogramming of stem cells 10, 11. However, its overexpression inhibits BRAC1 and BRCA2, which may repair DNA double strand breaks 12, 13. AURKA overexpression was also found to be significantly related to high-grade and high-stage hepatocellular carcinoma tumors 14. In addition, AUKRA amplification has been found in a variety of cancers, such as breast and ovarian cancers 15, 16.

If a single nucleotide difference in the shared sequence of a gene in a pair of chromosomes exceeds 1% of certain populations, it is defined as a single nucleotide polymorphism (SNP)^17^-^20^. When SNPs exhibit an influence on the promoter area, exon and 3'-untranslated region of a gene, it may affect the gene expression, thereby promote the occurrence of disease and cancer 17, 21-23. Numerous SNPs in AUKRA genes, including rs2273535, rs6024836, rs2064863 and rs1047972, have been identified as being associated with cancer progression and cancer susceptibility 24-27. To the best of our knowledge, no study investigates the relationships among AURKA genetic polymorphisms and cervical tumorigenesis in Taiwanese female individuals, as well as the clinicopathological characteristics and patient survival of cervical cancer. Therefore, we conducted this study to delineate the involvement of AURKA in the cervical carcinogenesis and clinical variables and patient survival of cervical cancer.

## Materials and methods

### Female individuals

The Department of Obstetrics and Gynecology of the Affiliated Hospital of Chung Shan Medical University in Taiwan enrolled 106 patients with invasive cancer and 94 patients with precancerous lesions of uterine cervix. Meanwhile, 302 female individuals without cervical lesions were included as a control group. All subjects lived in central Taiwan, where is our hospital. Therefore, the study population was recruited from this area. In our hospital, the incidence of cervical cancer is similar to other regions of Taiwan. Patients with cervical precancerous lesions or invasive cancer had undergone standard treatment protocols at the Department of Obstetrics and Gynecology in Chung Shan Medical University Hospital between January 2000 and December 2014. The clinical staging of cervical cancer patients was based on the 2009 International Federation of Obstetrics and Gynecology system. The official pathology report confirmed the diagnosis of cervical invasive carcinoma and high-grade CIN. Neither SIL lesions nor cancers cells were found in the cervical smear reports of control women. These smears examinations were done in the outpatient clinic of the Affiliated Hospital of Chung Shan Medical University, and the cytological diagnosis was further verified by colposcopy to meet the results of the smear examination. The study was conducted under the approval of the Institutional Review Board of the Chung Shan Medical University Hospital (CSMUH number: CS18208), and everyone's informed consent was obtained.

### Deoxyribonucleic acid (DNA) extraction from blood samples of all participants and selection of AURKA SNPs

Laboratory staff used venipuncture techniques to draw blood samples from all participants. The samples were collected in a Vacutainer tubes mixed with ethylenediaminetetraacetic acid. They were stored at 4 °C immediately. DNA was extracted using the QIAamp DNA Blood Mini Kit according to the manufacturer's instructions 28, 29. The recruited DNA was therefore dissolved in pH 7.8 TE buffer. Then, it was quantified by the measurement of OD260. The OD260/OD280 ratio was checked and the range of 1.8-2.0 met our standards and defined as pure to prevent it from cross-reacting with the current homologous RNA in the samples. The final product were then stored at -20 °C and used as a template for the polymerase chain reaction (PCR).

Based on the data of the international HapMap project and previous publication by Mesic et al., four AURKA SNPs were selected 30. AURKA genetic variants rs2273535, rs6024836, rs2064863 and rs1047972 were examined through ABI StepOne Real-Time PCR System (Applied Biosystems, Foster City, CA, USA), and determined with SDS vers. 3.0 software, as our previous publication 26.

### Statistical analysis

Analysis of variance (ANOVA) was used to compare the age distribution of the female individuals studied, and Scheffe test was applied for post hoc analysis. The Hardy-Weinberg equilibrium was performed to examine the genotypic frequencies of AURKA rs2273535, rs6024836, rs2064863 and rs1047972 in normal controls [degree of freedom (d.f.)=2].

In order to define the relationships between AURKA SNPs and cervical neoplasias, chi-square or Fisher's exact tests was done. The adjusted odds ratios (AORs) with their 95% confidence intervals (CIs) were calculated to check the relationships among genotypic distributions of AURKA SNPs and the incidence of cervical neoplasias (including precancerous lesions and invasive cancer) through the logistic and multinomial logistic regression models for controlling age of the subjects.

Chi-square or Fisher's exact tests were used to define the relationships among AURKA SNPs and clinicopathological parameters as well as recurrence and mortality events of cervical cancer. Kaplan-Meier curve model (univariate analysis over time) was used to determine the significance of AURKA SNPs and clinicopathological parameters in the prognoses of patients, which were statistically related to with recurrence-free and overall survival of cervical cancer patients. The log-rank test was tested to examine the differences among them. In the multivariate analysis of recurrence-free and overall survival time, a Cox proportional hazard model of a forward stepwise method was used to examine the influences of AURKA genetic variants and a variety of clinicopathological factors on recurrence-free and overall survival. Thus, the hazard ratios (HRs) were calculated. The statistical analysis of SPSS, version 18.0 and WinPepi Software, version 10.0 was done for statistical significance. *p*<0.05 was regarded as statistically significant difference.

## Results

It indicated that there was significant difference in the age distribution between patients with cervical neoplasias and normal control women (50.1 ± 14.0 vs. 43.8 ± 10.0, *p*<0.001). A post hoc analysis based on the Scheffe test of the ANOVA found that the age distribution between patients with cervical invasive cancer and those with precancerous lesions was statistically different (55.6 ± 12.8 vs. 43.9 ± 12.7, *p*<0.001), as well as statistically different between patients with cervical cancer and control women (55.6 ± 12.8 vs. 43.8 ± 10.0, *p*<0.001) but not statistically different between those with precancerous lesions and control women (43.9 ± 12.7 vs. 43.8 ± 10.0, *p*= 0.999).

The minor allele frequencies of AURKA genetic variants rs2273535, rs6024836, rs2064863 and rs1047972 in control female individuals were all ≥5%. Genotypic frequency of AURKA genetic variant rs2273535 conformed to the Hardy-Weinberg equilibrium [χ^2^ value, 0.114, *p*=0.945; d.f.=2] in control females. The frequencies of rs6024836, rs2064863 and rs1047972 were also satisfied the Hardy-Weinberg equilibrium (χ^2^ value, 3.976, *p*=0.137; χ^2^ value, 0.367, *p*=0.833 and χ^2^ value, 0.093, *p*=0.955; respectively).

Table 1 lists genetic polymorphism frequencies of AURKA gene in Taiwanese women with cervical neoplasias and normal controls. There were no significantly different in the distributions of AURKA SNPs rs2273535, rs6024836, rs2064863 and rs1047972 between patients with cervical neoplasias and normal control women. After controlling for age, there were still no different distributions between patients with cervical neoplasias and normal controls. After categorizing cervical neoplasias into precancerous lesions and invasive cancer subgroups, Table 2 reveals the genotypic frequencies of AURKA gene in patients with cervical invasive cancer and precancerous lesions as well as normal controls. Even after adjusting for age, there were no significant differences in the distributions of AURKA SNPs rs2273535, rs6024836, rs2064863 and rs1047972 among patients with cervical invasive cancer and precancerous lesions as well as normal control women.

When AURKA SNPs were related to the clinicopathological parameters, no statistical significances were found among them (Table 3). We further investigate the associations of AURKA SNPs with recurrence and survival events of cervical cancer patients. Neither AURKA rs6024836 nor other AURKA SNPs are associated with recurrence and mortality events of cervical cancer patients (Table 4). But, cervical cancer patients accompanied by adenocarcinoma, deeper stromal invasion, larger tumor diameter, positive vagina invasion and positive pelvic lymph node metastasis had a higher risk of recurrence event. Furthermore, patients with clinical stage ≥ stage II, deeper stromal invasion, larger tumor diameter, positive parametrium and vagina invasion as well as positive pelvic lymph nodes metastasis exhibited a higher risk of mortality event (Table 4). However, it did not reveal different distributions of rs2273535, rs6024836, rs2064863 and rs1047972 among patients with different clinical stage, different pathological type, stromal invasion depth, tumor size, parametrium and vagina invasive conditions and pelvic lymph node metastasis status of cervical cancer patients.

In the Kaplan-Meier curve univariate analysis model, AURKA rs6024836 was not related to recurrence-free survival and overall survival of cervical cancer patients (Table 5). Other AURKA SNPs also did not play any statistical significance in the prognoses of these patients. Cervical cancer patients, who exhibited clinical stage ≥ stage II, adenocarcinoma, deeper stromal invasion, larger tumor diameter, positive vagina invasion and positive pelvic lymph node metastasis, had poorer recurrence-free survival. Cervical cancer patients with ≥ stage II clinical stage, deeper stromal invasion, larger tumor diameter, positive parametrium and vagina invasions and positive pelvic lymph node metastasis had poorer overall survival (Table 5).

In a multivariate analysis using Cox proportional hazard model to adjust AURKA SNPs and various clincopathological factors, neither AURKA rs6024836 nor other AURKA SNPs were showed to be associated with recurrence-free survival and overall survival (Table 6). However, cervical cancer patients with adenocarcinoma (HR: 3.18, 95% CI: 1.23-8.23; *p*=0.017) and larger tumor (HR: 5.61, 95% CI: 2.10-14.95;* p*=0.001) had poorer recurrence-free survival. Those with deeper stromal invasion (HR: 3.52, 95% CI: 1.22-10.12;* p*=0.020) and positive pelvic lymph node metastasis (HR: 4.42, 95% CI: 1.82-10.71;* p*=0.001) had poorer overall survival (Table 6).

## Discussion

It was reported that AURKA was related to various cancers such as colorectal and pancreatic cancers 31, 32. Genetic polymorphisms may display an influence on the gene expression by affecting promoter regions, exons introns, and 3'-untranslated regions 17, 21, 22. Elevated AURKA expression might cause chromosomal instability and centrosome amplification in mammalian cells 33. Genetic instability, probably leading to mutations of oncogenes and tumor-suppressor genes, is an important driving forces of malignant transformation and cancer progression 34. AURKA overexpression may promote c-Myc oncogenic effects and cause its increased expression 35, which is associated with the development of a variety of cancers, including gynecological malignancies such as ovarian, endometrial and cervical cancers 36-38.

To date, no reports have investigated the relationships between AURKA SNPs and the development of cervical cancer. Therefore, we conducted this study, and found no different frequencies of AURKA rs2273535, rs6024836, rs2064863 and rs1047972 between patients with cervical neoplasias and normal controls. After dividing cervical neoplasias into invasive cancer and precancerous subgroups, AURKA SNPs still did not participate in the occurrence of cervical cancer. AURKA SNP rs2273535 is situated at nucleotide position 91 of exon 4 of chromosome 20q13.2 and is a non-synonymous polymorphism, encoding a phenylalanine to isoleucine substitution at amino acid position 31. Moreover, Wang et al. conducted a systemic review through the meta-analysis to reveal the involvement of AURKA rs2273535 in cancer risk and demonstrated that allele change from T to A in rs2273535 was related to an overall increased risk of cancers, especially for breast cancer 39. rs1047972 is another nonsynonymous variant, which is also situated at exon 4, and leads to a valine to isoleucine substitution at amino acid position 57 40. Although AURKA SNPs may have an impact on amino acid changes, this study did not seem to show that AURKA SNPs have an influence on the development of cervical cancer. Ishikawa et al. found that an allele T in AURKA SNP rs2273535 and T in rs1047972 were correlated with a reduced early adverse reaction in cervical cancer patients who received pelvic radiotherapy, but this has nothing to do with the cancer itself 41. In addition, Mesic et al. suggested that AURKA rs1047922 polymorphisms may influence the development of gastric cancer 42. Although AURKA SNP rs758099 is located in intron area, they also found that it could have an impact on gastric cancer development.^30^ Interestingly, Dai el al. used meta-analysis to indicate that AURKA SNP rs2273535 increased the risk of breast cancer especially among Asians, whereas, rs1047972 SNP reduced the risk of breast cancer in Caucasians 43.

Taking into account the relationships between AURKA SNPs and clinicopathological characteristics of cervical cancer, it showed that AURKA SNPs were not associated with these parameters. Cervical cancer patients with clinical stage ≥ stage II, deeper stromal invasion, larger tumor diameter, positive parametrium and vagina invasions as well as positive pelvic lymph nodes metastasis were at higher risk of mortality event. Since AURKA SNPs are not associated with these parameters, it is reasonable that AURKA SNPs are not linked to recurrence and mortality events in this study. Moreover, Chou et al. suggested that AURKA SNP rs2064863 was related to more advanced stages (stages III/IV) in patients with oral squamous cell carcinoma 24. In contrast, rs2064863 was not concerned with tumor size, regional lymph node metastasis and distal organs metastases as well as cell differentiation. Therefore, we subdivide the stage classification of cervical cancer patients into stage I/II subgroup and III/IV subgroup to associate AURAA SNPs with cancer stages. But, it still fails to draw a significant relationship between AURKA and cancer stages (data not shown). However in hepatocellular carcinoma (HCC), Wang et al. demonstrated that patients, who carried at least one allele A in AURKA SNP rs2273535, were less likely to develop into stage III/IV disease and have large tumor. In addition, patients, who carried at least one allele G in rs2064863, exhibited a lower risk of developing large tumors. It indicated that AURKA SNPs could predict early-stage HCC 26.

When time interval was included to correlate AURKA SNPs with prognosis, recurrence-free survival and overall survival were assessed in cervical cancer patients. In univariate Kaplan-Meier curve analysis, there were there no statistical significances between AURKA SNPs and recurrence-free survival, and overall survival. Furthermore in multivariate Cox proportional hazard model analysis, there were still no differences between AURKA SNPs and these patient prognoses. So far, no study explores the relationship of AURKA SNPs and the survival of cervical cancer patients. However in triple-negative breast cancer patients, Liao et al. found that AURKA SNPs may predict overall survival and disease-free survival 44. Finally, we found that tumor diameter was the most obvious independent predictor of recurrence-free survival in cervical cancer patients. However, pelvic lymph node metastasis was the most obvious predictor in assessing overall survival in cervical cancer patients. It is rational because previous investigation has found that pelvic lymph node metastasis was critical in predicting patient survival of cervical cancer 45, 46.

In conclusion, AURKA SNPs rs2273535, rs6024836, rs2064863 and rs1047972 does not seem to be involved in the occurrence of cervical cancer in Taiwanese women. They are also not related to the clinicopathological variables of cervical cancer. These SNPs cannot predict the prognosis of cervical cancer patients. Because the studied sample was relatively small and human papillomavirus infection status lacked because of conservative attitude of Taiwanese women, it is necessary to conduct further research with a larger sample size to draw a definite conclusion.

## Figures and Tables

**Table 1:** Distributions of genetic polymorphism of aurora kinase A gene in women with uterine cervical neoplasias and normal controls in Taiwan

Genetic polymorphisms	Normal controls (n =302)	Cervical neoplasias^a^ (n=200)	ORs (95% CIs)	*p* values	AORs (95% CIs)^b^	Adjusted *p* values^b^
**rs2273535**				0.730		0.936
TT^c^	138	96	1.00		1.00	
TA	134	88	0.94 (0.65-1.37)	0.763	0.97 (0.66-1.43)	0.869
AA	30	16	0.77 (0.40-1.48)	0.430	0.88 (0.45-1.74)	0.721
TT^c^	138	96	1.00	0.612	1.00	0.800
TA/AA	164	104	0.91 (0.64-1.30)		0.95 (0.66-1.38)	
TT/TA^c^	272	184	1.00	0.463	1.00	0.746
AA	30	16	0.79 (0.42-1.49)		0.90 (0.47-1.72)	
**rs6024836**				0.855		0.543
AA^c^	120	78	1.00		1.00	
AG	152	99	1.00 (0.68-1.47)	0.992	1.08 (0.73-1.61)	0.689
GG	30	23	1.18 (0.64-2.18)	0.598	1.43 (0.76-2.70)	0.269
AA^c^	120	78	1.00	0.869	1.00	0.506
AG/GG	182	122	1.03 (0.72-1.49)		1.14 (0.78-1.66)	
AA/AG^c^	272	177	1.00	0.576	1.00	0.302
GG	30	23	1.18 (0.66-2.09)		1.37 (0.76-2.48)	
**rs2064863**				0.965		0.930
TT^c^	203	135	1.00		1.00	
TG	91	59	0.98 (0.66-1.45)	0.899	1.01 (0.67-1.52)	0.953
GG	8	6	1.13 (0.38-3.32)	0.827	1.24 (0.41-3.79)	0.704
TT^c^	203	135	1.00	0.948	1.00	0.881
TG/GG	99	65	0.99 (0.67-1.45)		1.03 (0.69-1.53)	
TT/TG^c^	294	194	1.00	0.815	1.00	0.707
GG	8	6	1.14 (0.39-3.33)		1.24 (0.41-3.75)	
**rs1047972**				0.810		0.898
CC^c^	240	161	1.00		1.00	
CT	59	36	0.91 (0.57-1.44)	0.686	0.93 (0.58-1.50)	0.769
TT	3	3	1.49 (0.30-7.48)	0.628	1.33 (0.26-6.82)	0.733
CC^c^	240	161	1.00	0.778	1.00	0.838
CT/TT	62	39	0.94 (0.60-1.47)		0.95 (0.60-1.51)	
CC/ CT^c^	299	197	1.00	0.612	1.00	0.720
TT	3	3	1.52 (0.30-7.60)		1.35 (0.26-6.89)	

Statistical analysis: logistic regression model or chi-square or Fisher's exact tests; ^a^Cervical neoplasias consist of precancerous lesions and invasive cancer of the uterine cervix; ^b^The adjusted *p* values and adjusted odds ratios, and their 95% confident intervals were determined by logistic regression model after controlling age; ^c^Used as a reference for comparison to determine the odds ratios of other genotypes. 95% CIs, 95% confidence intervals.

**Table 2:** Distributions of genetic polymorphism of aurora kinase A gene in women with uterine cervical invasive cancer or precancerous lesions and normal controls in Taiwan

Genetic polymorphisms	Normal controls (n =302)	Pre-cancerous lesions (n =94)	Invasive cancer (n =106)	*p* values	AORs (95% CIs)^a^	Ad. *p* values	AORs (95% CIs)^b^	Ad. *p* values
**rs2273535**								
TT^c^	138	42	54	0.237	1.00		1.00	
TA	134	40	48		0.98 (0.60-1.61)	0.941	0.93 (0.57-1.54)	0.783
AA	30	12	4		1.32 (0.62-2.80)	0.476	0.44 (0.14-1.37)	0.157
TT^c^	138	42	54	0.594	1.00		1.00	
TA/AA	164	52	52		1.04 (0.65-1.66)	0.861	0.85 (0.52-1.38)	0.512
TT/TA^c^	272	82	102	0.068	1.00		1.00	
AA	30	12	4		1.33 (0.65-2.71)	0.436	0.46 (0.15-1.38)	0.165
**rs6024836**								
AA^c^	120	32	46	0.162	1.00		1.00	
AG	152	46	53		1.14 (0.68-1.90)	0.621	1.04 (0.63-1.73)	0.867
GG	30	16	7		2.01 (0.98-4.14)	0.059	0.83 (0.32-2.14)	0.703
AA^c^	120	32	46	0.396	1.00		1.00	
AG/GG	182	62	60		1.28 (0.79-2.08)	0.319	1.01 (0.62-1.66)	0.956
AA/AG^c^	272	78	99	0.049	1.00		1.00	
GG	30	16	7		1.86 (0.97-3.60)	0.063	0.81 (0.33-2.00)	0.651
**rs2064863**								
TT^c^	203	63	72	0.896	1.00		1.00	
TG	91	27	32		0.96 (0.57-1.60)	0.867	1.03 (0.60-1.76)	0.909
GG	8	4	2		1.61 (0.47-5.53)	0.448	0.78 (0.15-4.12)	0.772
TT^c^	203	63	72	0.989	1.00		1.00	
TG/GG	99	31	34		1.01 (0.62-1.65)	0.969	1.01 (0.60-1.70)	0.969
TT/TG^c^	294	90	104	0.599	1.00		1.00	
GG	8	4	2		1.63 (0.48-5.55)	0.431	0.78 (0.15-4.05)	0.763
**rs1047972**								
CC^c^	240	72	89	0.291	1.00		1.00	
CT	59	19	17		1.07 (0.60-1.92)	0.811	0.79 (0.41-1.50)	0.463
TT	3	3	0		3.34 (0.66-17.01)	0.146	u.a.	u.a.
CC^c^	240	72	89	0.414	1.00		1.00	
CT/TT	62	22	17		1.18 (0.68-2.06)	0.552	0.73 (0.39-1.38)	0.335
CC/ CT^c^	299	91	106	0.084	1.00		1.00	
TT	3	3	0		3.30 (0.65-16.70)	0.150	u.a.	u.a.

^a^Adjusted *p* values and adjusted odds ratios with their 95% CIs were determined using multinomial logistic regression models after controlling age between patients with uterine cervical precancerous lesions and control women; ^b^Adjusted *p* values and adjusted odds ratios with their 95% CIs were determined using multinomial logistic regression models after controlling age between patients with uterine cervical invasive cancer and control women; ^c^Used as a reference for comparison to calculate the odds ratios of other genotypes; AORs, adjusted odds ratios; 95% CIs, 95% confidence intervals; Ad. *p*, adjusted *p*; u.a., unavailable.

**Table 3:** Relationships between genotypic distribution of aurora kinase A rs6024836 and clinicopathological parameters in patients with cervical invasive cancer

Parameters^a^	rs6024836		*p* value	ORs; 95% CIs
	AA/AG^b^	GG		
**Clinical stage**			0.136	1.00; 0.20 (0.02-1.72)
stage I^b^	54	6		
≥ stage II	45	1		
**Pathologic type**			1.000	1.00; u.a.
squamous cell carcinoma^b^	89	7		
adenocarcinoma	10	0		
**Cell grading**			0.093	1.00; 0.24 (0.05-1.17)
well (grade 1)^b^	15	3		
moderate & poor (grades 2/3)	84	4		
**Stromal invasion depth**			0.118	1.00; 0.17 (0.02-1.50)
≤10 mm^b^	50	6		
>10 mm	48	1		
**Tumor diameter**			0.124	1.00; 0.18 (0.02-1.59)
≤4 cm^b^	52	6		
>4 cm	47	1		
**Parametrium**			0.250	1.00; 0.26 (0.03-2.21)
no invasion^b^	60	6		
invasion	39	1		
**Vagina**			0.237	1.00; 0.23 (0.03-1.95)
no invasion^b^	57	6		
invasion	42	1		
**Pelvic lymph node**			0.671	1.00; 0.38 (0.04-3.32)
no metastasis^b^	69	6		
metastasis	30	1		

Statistical analyses: chi-square or Fisher's exact tests; ^a^Some clinicopathological data could not be recruited from all the patients with cervical invasive cancer due to incomplete medical charts or records; ^b^As a reference. ORs, odds ratios; 95% CIs, 95% confidence intervals; u.a., unavailable.

**Table 4:** Analysis of aurora kinase A (AURKA) genetic variants and clinicopathological characteristics in recurrence or mortality events of cervical cancer patients

Variables^a^	Recurrence (N= 106)				Mortality (N=106)			
	+	-	*p* value	ORs (95% CIs)	+	-	*p* value	ORs (95% CIs)
**AURKA rs6024836**			1.000				1.00	
AA/AG^b^	24	73		1.00	77	25		1.00
GG	1	5		0.61 (0.07-5.47)	1	0		u.a.
**Clinical stage**			0.025				<0.001	
stage I^b^	9	48		1.00	51	6		1.00
≥ stage II	16	30		2.84 (1.12-7.25)	27	19		5.98 (2.14-16.75)
**Pathologic type**			0.012				0.702	
squamous cell carcinoma^b^	19	74		1.00	71	22		1.00
adenocarcinoma	6	4		5.84 (1.50-22.81)	7	3		1.38 (0.33-5.81)
**Cell grading**			0.755				0.755	
well (grade 1)^b^	3	13		1.00	13	3		1.00
moderate & poor (grades 2/3)	22	65		1.47 (0.38-5.63)	65	22		1.47 (0.38-5.63)
**Stromal invasion depth**			0.001				<0.001	
≤10 mm^b^	6	47		1.00	48	5		1.00
>10 mm	19	30		4.96 (1.78-13.84)	29	20		6.62 (2.24-19.55)
**Tumor diameter**			0.001				<0.001	
≤4 cm^b^	6	49		1.00	50	5		1.00
>4 cm	19	29		5.35 (1.92-14.93)	28	20		7.14 (2.42-21.11)
**Parametrium**			0.121				0.001	
no invasion^b^	12	51		1.00	55	8		1.00
invasion	13	27		2.05 (0.82-5.10)	23	17		5.08 (1.92-13.42)
**Vagina**			0.033				0.033	
no invasion^b^	10	50		1.00	50	10		1.00
invasion	15	28		2.68 (1.06-6.75)	28	15		2.68 (1.06-6.75)
**Pelvic lymph node**			0.006				<0.001	
no metastasis^b^	12	60		1.00	64	8		1.00
metastasis	13	18		3.61 (1.40-9.29)	14	17		9.71 (3.50-26.94)

Statistical analysis: Chi-square or Fisher's exact tests; ^a^Some clinicopathological data could not be recruited from all patients with cervical invasive cancer due to incomplete records of medical chart; ^b^As a reference; Recurrence: +, recurrence; -, no recurrence. Survival: +, survival; -, mortality. u.a.: unavailable.

**Table 5:** Univariate analysis of the associations among aurora kinase A (AURKA) genetic polymorphism rs6024836 and various clinicopatholgical factors and the recurrence-free and overall survival of the patients with uterine cervical cancer

Variables	Recurrence-free survival		Overall survival	
	*p* value	HR & 95% CI^b^	*p* value	HR & 95% CI^b^
**AURKA rs6024836**				
GG vs AA/AG^a^	0.656	0.67 (0.09-4.98)	0.656	0.68 (009-5.04)
***Clinicopathological characteristics***				
**Stage**				
≥ stage II vs stage I^a^	0.026	2.20 (0.97-4.99)	<0.001	3.64 (1.44-9.22)
**Pathologic type**				
Squamous cell carcinoma^a^ adenocarcinoma	0.006	2.94 (1.17-7.35)	0.658	1.53 (0.45-5.16)
**Cell grading**				
Well (grade 1)^a^; moderate & poor (grade 2/3)	0.577	1.35 (0.40-4.51)	0.577	1.49 (0.45-4.98)
**Stromal invasion depth**				
>10 mm vs ≤10 mm^a^	0.001	3.43 (1.37-8.58)	<0.001	5.46 (2.03-14.67)
**Tumor diameter**				
>4 cm vs ≤ 4cm^a^	0.001	3.63 (1.45-9.09)	<0.001	5.36 (2.01-14.31)
**Parametrium**				
invasion vs no invasion^a^	0.122	1.71 (0.78-3.74)	0.001	3.37 (1.45-7.81)
**Vagina**				
invasion vs no invasion^a^	0.034	2.09 (0.94-4.66)	0.034	2.18 (0.98-4.88)
**Pelvic lymph node**				
metastasis vs no metastasis^a^	0.006	2.52 (1.15-5.51)	<0.001	6.51 (2.80-15.17)

Statistical analyses: Kaplan-Meier curves model; ^a^As a comparison reference; ^b^HR, hazard ratio and 95% CI, 95% confidence interval for AURKA genetic variant rs6024836 and clinicopathological variables, compared with their respective controls.

**Table 6:** Multivariate analysis of the associations among aurora kinase A gene (AURKA) genetic polymorphism rs6024836 and various clinicopatholgical factors and the recurrence-free and overall survival of the patients with uterine cervical cancer

Variables	Recurrence-free survival		Overall survival	
	*p* value	HR & 95% CI^b^	*p* value	HR & 95% CI^b^
**AURKA rs6024836**				
GG vs AA/AG^a^	0.472	2.18 (0.26-18.26)	0.787	0.76 (0.10-5.68)
**Pathologic type**				
Squamous cell carcinoma^a^ adenocarcinoma	0.017	3.18 (1.23-8.23)	insignificant	1.23 (0.34-4.45)
**Stromal invasion depth**				
>10 mm vs ≤10 mm^a^	insignificant		0.020	3.52 (1.22-10.12)
**Tumor diameter**				
>4 cm vs ≤ 4cm^a^	0.001	5.61 (2.10-14.95)	insignificant	1.82 (0.42-7.97)
**Pelvic lymph node**				
metastasis vs no metastasis^a^	insignificant	2.02 (0.79-5.17)	0.001	4.42 (1.82-10.71)

Statistical analyses: Cox proportional hazard model with a forward stepwise approach; ^a^As a comparison reference; ^b^HR, hazard ratio and 95% CI, 95% confidence interval for AURKA genetic variant rs6024836 and clinicopathological variables, compared with their respective controls.
